# Chemerin Regulates the Proliferation and Migration of Pulmonary Arterial Smooth Muscle Cells *via* the ERK1/2 Signaling Pathway

**DOI:** 10.3389/fphar.2022.767705

**Published:** 2022-03-18

**Authors:** Linqian Peng, Yunwei Chen, Yan Li, Panpan Feng, Yan Zheng, Yongjie Dong, Yunjing Yang, Ruiyu Wang, Ailing Li, Jianghong Yan, Feifei Shang, Ping Tang, Dewei Chen, Yuqi Gao, Wei Huang

**Affiliations:** ^1^ Department of Cardiology, The First Affiliated Hospital of Chongqing Medical University, Chongqing, China; ^2^ Institute of Life Science, Chongqing Medical University, Chongqing, China; ^3^ Institute of Medicine and Equipment for High Altitude Region, College of High Altitude Military Medicine, Army Medical University, Chongqing, China

**Keywords:** chemerin, chemokine-like receptor 1, pulmonary arterial hypertension, pulmonary arterial smooth muscle cells, ERK1/2

## Abstract

Pulmonary arterial hypertension (PAH) is an incurable disease with high mortality. Chemerin has been found to be associated with pulmonary hypertension (PH). However, the specific role of chemerin in mediating PH development remains unclear. This study aimed to elucidate the regulatory effects and the underlying mechanism of chemerin on PH and to investigate the expression levels of chemerin protein in plasma in PAH patients. *In vivo*, two animal models of PH were established in rats by monocrotaline (MCT) injection and hypoxia. We found that the expression levels of chemerin and its receptor, chemokine-like receptor 1 (CMKLR1), were significantly upregulated in the lungs of PH rats. Primary cultured pulmonary arterial smooth muscle cells [(PASMCs) (isolated from pulmonary arteries of normal healthy rats)] were exposed to hypoxia or treated with recombinant human chemerin, we found that CMKLR1 expression was upregulated in PASMCs in response to hypoxia or chemerin stimulation, whereas the exogenous chemerin significantly promoted the migration and proliferation of PASMCs. Notably, the regulatory effects of chemerin on PASMCs were blunted by PD98059 (a selective ERK1/2 inhibitor). Using enzyme linked immunosorbent assay (ELISA), we found that the protein level of chemerin was also markedly increased in plasma from idiopathic pulmonary arterial hypertension (IPAH) patients compared to that from healthy controls. Moreover, the diagnostic value of chemerin expression in IPAH patients was determined through receiver operating characteristic (ROC) curve analysis and the result revealed that area under ROC curve (AUC) for plasma chemerin was 0.949. Taken together, these results suggest that chemerin exacerbates PH progression by promoting the proliferation and migration of PASMCs via the ERK1/2 signaling pathway, and chemerin is associated with pulmonary hypertension.

## Introduction

As a serious life-threatening disease, pulmonary arterial hypertension (PAH) is characterized by pulmonary vascular remodeling, which often leads to progressive increases in pulmonary arterial pressure (PAP) and pulmonary vascular resistance (PVR), eventually resulting in right heart failure and death (Rich and Rich 2014). The primary pathological signs of pulmonary vascular remodeling include media hypertrophy, intimal proliferation, and adventitial thickening and fibrosis (Tuder 2017). Of note, among them, the proliferation of pulmonary arterial smooth muscle cells (PASMCs) is the main pathological feature of pulmonary vascular remodeling (Morrell et al., 2009). Mechanistically, inappropriate activation of the ERK1/2 signaling pathway plays a crucial role in the regulation of pulmonary vascular remodeling, and suppressing ERK1/2 activation effectively delays PH progression (Yu et al., 2017; Xing et al., 2019; Yang et al., 2021).

Chemerin, which is encoded by retinoic acid receptor responder 2 (RARRES2), is an adipokine secreted by the liver and adipocytes and is closely associated with inflammation, adipocyte growth, angiogenesis, and energy metabolism (Kennedy and Davenport 2018). Chemerin can bind to the following three receptors: chemokine-like receptor 1 (CMKLR1), C-C motif chemokine receptor like 2 (CCRL2) and G protein-coupled receptor 1 (GPR1) (Kennedy and Davenport 2018). Among them, CCRL2 elevates the local concentration of chemerin to promote chemerin binding to GPR1 and CMKLR1 (Zabel et al., 2008). Although GPR1 may be associated with glucose homeostasis, little is known about its specific biological effects (Rourke et al., 2014). In fact, chemerin participates in a variety of biological processes mainly by activating CMKLR1 (Kennedy et al., 2016; Wen et al., 2019). Therefore, the chemerin/CMKLR1 axis plays an important role in tumorigenesis, cardiovascular disease, metabolic syndrome and other conditions (Kunimoto et al., 2015; Weng et al., 2017; Jiang et al., 2018; Shin and Pachynski 2018). Furthermore, a study on systemic hypertension demonstrated that the chemerin/CMKLR1 axis regulated the activation and transduction of ERK1/2 signaling during vascular remodeling (Kunimoto et al., 2015), which has led to interest in exploring the association between chemerin and PH.

A recent study showed that chemerin and its receptor (CMKLR1) could be detected in the pulmonary arterial tissues of rats (Hanthazi et al., 2020). Chemerin-9-induced contraction was enhanced through the upregulation of CMKLR1 in the isolated pulmonary arteries of PH rats (Omori et al., 2020). Recombinant chemerin protein exogenously enhanced the effect of endothelin (ET) on the proliferation and migration of PASMCs (Hanthazi et al., 2020); however, chemerin alone failed to promote the proliferation of PASMCs directly. Accordingly, the specific role of chemerin in mediating PH has not been fully elucidated, and the expression levels of chemerin protein in plasma in PAH patients was not known.

In the present study, we examined alterations in the chemerin/CMKLR1 axis both in monocrotaline (MCT)/hypoxia induced PH rats and in hypoxia-treated PASMCs. Moreover, the effects of chemerin on pulmonary vascular remodeling and the underlying mechanisms in PASMCs were also explored using recombinant chemerin protein. Furthermore, expression levels of chemerin protein in the plasma of idiopathic pulmonary arterial hypertension (IPAH) patients were determined and the diagnostic value of chemerin expression in IPAH patients was estimated using receiver operating characteristic (ROC) curve analysis.

## Materials and Methods

### Study Population

This clinical study was approved by the Ethics Committee of the First Affiliated Hospital of Chongqing Medical University. Written informed consent was obtained from all the participants. In this study, control subjects (*n* = 21) were recruited from healthy volunteers, and IPAH patients (*n* = 14) were enrolled at the First Affiliated Hospital of Chongqing Medical University from april 2016 to December 2020. IPAH was diagnosed according to the following criteria: mean pulmonary arterial pressure (mPAP) > 20 mmHg, pulmonary artery wedge pressure (PAWP) ≤ 15 mmHg and PVR ≥3 Wood units measured at rest and at sea level by right heart catheterization (RHC) (Rosenkranz et al., 2019). No etiology or familial history of PAH was identified in IPAH patients. All clinical data and samples were collected during the patient admission at the first visit. Plasma samples were collected as follows: whole blood was collected and centrifuged (3,000 rpm, 10 min), and plasma was collected and stored at −80°C.

### Enzyme Linked Immunosorbent Assay

Plasma levels of chemerin were measured using ELISA kit (Ruixin Biotech, Quanzhou, China). Blank control, standard and sample wells were set following the manufacturer’s instructions. Then, each well was added with HRP-labeled antibody (100 μL/well) followed by shaking gently. After being incubated at 37°C for 30 min, the reaction plates were rinsed repeatedly with a washing liquor. Next, 100 μL of substrate mixture was added to each well and incubated at 37°C for 15 min. Finally, 50 μL of stop solution was added per well and the absorbance was read at 450 nm wavelength. The plasma concentrations of chemerin were calculated using the standard curve.

### Animal Experiments

Specific pathogen-free (SPF) male Sprague-Dawley (SD) rats (*n* = 20,180–200 g) were purchased from the Experimental Animal Center of Chongqing Medical University (Chongqing, China) and raised in individual ventilated cages (IVCs) in the animal experiment room. All experiments were conducted in accordance with the guidelines of the Ethics Committee of Chongqing Medical University and in compliance with the Guidelines for the Care and Use of Laboratory Animals published by the National Institutes of Health (Bethesda, MD, United States). Twenty SD rats were randomly divided into the control and monocrotaline (MCT, Mengbio, Chongqing, China) groups (*n* = 10 per group). MCT (500 mg) was dissolved in anhydrous alcohol (10 ml) and saline (40 ml). Rats in the MCT group were intraperitoneally (i.p.) injected with MCT (55 mg/kg), and the remaining rats were injected with equal volumes of a mixture of alcohol and saline (1:4) as controls. And other twelve rats were randomly divided into the nomoxia and hypoxia groups (*n* = 6 per group). Rats in the hypoxia group were housed in a closed chamber with 12% oxygen concentration for 4 weeks to induce PH and there was no intervention in the control rats.

### Echocardiography

On the 21st day after MCT injection, the rats were anesthetized with 2% pentobarbital sodium (Merck KGaA; 60 mg/kg, i. p.), and the fur on the left chest was removed with depilatory cream. Then, echocardiography was performed with an ultrasound system (IE33, Philip, Holland) to examine right ventricular structure and function.

### RHC

Hemodynamic data of rats were measured by RHC. A polyethylene catheter (inner diameter, 0.5 mm; outside diameter, 0.9 mm) filled with heparinized saline was connected to the MP150 multi-channel physiological recorder (BIOPAC Systems, CA, United States). Subsequently, the anesthetized rats were fixed on the operating table and the right external jugular vein was exposed through surgery. Finally, the catheter was inserted into the right ventricle (RV) by cutting a V-shaped incision on the external jugular vein and introduced into pulmonary artery (PA) under the guidance of the pressure waveforms. The right ventricular systolic pressure (RVSP) and mPAP were measured by recorded waveforms of RV and PA, respectively.

### Histochemistry

Rat pulmonary tissues were fixed with 4% paraformaldehyde at 4°C, embedded in paraffin, and sectioned at a thickness of 5 µm for hematoxylin-eosin (HE) staining (Servicebio, Wuhan, China). Images of pulmonary arterioles were captured with a microscope (Leica Microsystems DFC550, Germany).

### Immunofluorescent Staining

Paraffin section of lung tissue were dewaxed and progressively rehydrated in alcoholic baths. Nonspecific binding sites were blocked with 3% bovine serum albumin (BSA) (Servicebio, Wuhan, China); After overnight incubation with a polyclonal rabbit anti-rat CMKLR1 (Affinity Antibodies, United States) or a polyclonal rabbit anti-rat chemerin (Invitrogen Antibodies, United States) and a polyclonal rabbit anti-rat α-SMA (Boster, Wuhan, China), sections were incubated for 50 min with corresponding secondary fluorescent-labeled antibodies [Goat Anti-Rabbit IgG(H + L)-Cy3, AIFang biological, Changsha, China]. Nuclei were counterstained with 4’,6-diamidino-2-phenylindole[(DAPI) (Servicebio, Wuhan, China)]. Images were taken using a fluorescence microscope (Leica Microsystems DFC550, Germany).

### Immunohistochemistry Staining

The lung tissue sections were deparaffinized and rehydrated in graduated alcohol. The endogenous peroxidase activity was blocked by incubation with 0.3% hydrogen peroxide at room temperature for 25 min. Then the sections were blocked with 3% bovine serum albumin (BSA) (Servicebio, Wuhan, China) for 30 min and then incubated with a polyclonal rabbit anti-rat α-SMA (Boster Antibodies, Wuhan, China) antibodies overnight, followed by incubation with the secondary antibody [Goat Anti-Rabbit IgG(H + L)-HRP, AIFang biological, Changsha, China] for 50 min. After the colour development through incubation with diaminobenzidine, the sections were counterstained with haematoxylin. The developed tissue sections were imaged under a microscope (Leica Microsystems DFC550, Germany).

### Isolation and Identification of PASMCs

Primary PASMCs were obtained from SD rats (6–8 weeks, 180–200 g) as previously described (Tang et al., 2020). Briefly, the lungs were rapidly excised after the rats were anesthetized with 2% pentobarbital sodium (Merck KGaA; 120 mg/kg, i. p.). The pulmonary arteries were isolated from the lungs in phosphate-buffered saline (PBS, HyClone, Utah, United States) and cut along the long axis of the vessel with ophthalmic scissors. Then, the endothelial cells and adventitial fibroblasts were gently removed by rubbing the internal and external surfaces of the pulmonary artery with tweezers. Subsequently, the remaining smooth muscle layer was cut into pieces, transferred to a culture dish with Dulbecco’s modified Eagle’s medium/nutrient mixture F-12 (DMEM/F-12, HyClone, Utah, United States) containing 20% fetal bovine serum (FBS, PAN-Biotech, Adenbach, Germany), and cultured in a humidified incubator at 37°C with 5% CO_2_. The purity of PASMCs was identified by immunofluorescence staining with an α-SMA antibody. Cells at passages 3-6 were used for subsequent experiments.

### Quantitative Reverse Transcription PCR

Total RNA was extracted from rat lung tissue with TRIzol reagent (Invitrogen, CA, United States). Then, cDNA was prepared by reverse transcription using the PrimeScript RT reagent kit with gDNA Eraser (TaKaRa, Japan) according to the manufacturer’s instructions. Subsequently, real-time quantitative PCR was conducted using SYBR^®^ Premix Ex Taq (TaKaRa, Japan) on an ABI7500 quantitative PCR instrument. PCR primers were designed using NCBI Primer-BLAST and synthesized by Tsingke (Chongqing, China). The sequences of primers used in the present study were as follows: Chemerin, 5′-AGG​GCC​TCT​CTA​AAG​CAA​CGA-3’ (Forward), 5′-CAA​GCT​CTG​TCC​CGT​GTA​TGT-3’ (Reverse); CMKLR1, 5′-TGT​GCT​TCC​TCG​GGA​TCC​TA-3’ (Forward), 5′-GGT​GAT​GTG​GAT​GGG​CAA​GA-3’ (Reverse); and β-actin, 5′-TCA​GGT​CAT​CAC​TAT​CGG​CAA​T-3’ (Forward), 5′-ACT​GTG​TTG​GCA​TAG​AGG​TCT​T-3’ (Reverse). The relative mRNA expression was calculated by the 2^-△△Ct^ method. β-actin was used as an internal control for normalizing the expression of the target genes.

### Western Blotting

Proteins were extracted from rat pulmonary tissues and PASMCs in RIPA lysis buffer with PMSF on ice. The protein concentration was determined by a BCA protein assay kit (MultiSciences, Hangzhou, China). Protein samples (15–25 μg) were separated by 12% SDS-PAGE and then transferred onto PVDF membranes (Bio-Rad, California, United States). After being blocked with 5% nonfat milk at room temperature for 1.5 h, the membranes were incubated with primary antibodies overnight at 4°C. Then, the membranes were incubated with horseradish peroxidase (HRP)-conjugated secondary antibodies (MultiSciences, Hangzhou, China) at room temperature for 1 h. Immunoreactive bands were visualized using an enhanced ECL kit (Beyotime, Shanghai, China). GAPDH,α-Tubulin or β-actin served as an endogenous control for equal sample loading. The primary antibodies used in this study were as follows: Chemerin (Invitrogen, CA, United States), CMKLR1 [(Bauer et al., 2011) (Abcam, Cambridge, UK)], proliferating cell nuclear antigen (PCNA) [(Hu et al., 2019) (Proteintech, Wuhan, China)], p-ERK1/2 (Thr202/Tyr204)[ (Karadeniz et al., 2021) (Cell Signaling Technology, Massachusetts, United States)], ERK1/2 [(Karadeniz et al., 2021) (Cell Signaling Technology, Massachusetts, United States)], β-actin [(Zhao et al., 2018) (Proteintech, Wuhan, China)], α-Tubulin [(Zhang et al., 2019) (Proteintech, Wuhan, China) ]and GAPDH [(Zhang et al., 2018) (Proteintech, Wuhan, China)].

### EdU Staining

A BeyoClick™ EdU-555 Cell Proliferation Kit (Beyotime Bio, Shanghai, China) was used for the cell proliferation assay. The PASMCs were incubated with10 μmol/L EdU for 2 h at 37 °C after treatment. The cells were permeabilized with Enhanced Immunostaining Permeabilization Buffer for 15 min after fixation with 4% formaldehyde for 15 min at room temperature. After three washes with PBS, the cells were incubated with click additive solution for 30 min. PASMC nuclei stained with Hoechst-33,342 were used for the cell counts and examined using fluorescence microscope.

### Scratch Wound Healing Assay

PASMCs at 90% confluence were wounded with a 200 µL pipette tip in 6-well plates. Then, the cells were washed three times to remove cell debris, pretreated with or without PD98059 (MCE, New Jersey, United States) for 30 min and cultured with recombinant human chemerin (R&D, Minneapolis, United States) as indicated. Wound closure was observed and photographed at 0 and 12 h with a microscope (Leica Microsystems DFC550, Germany).

### Transwell Migration Assay

The Transwell migration assay was performed in 24-well plates with Transwell chambers (8-μm pores; Biofil, Guangzhou, China). Briefly, PASMCs were trypsinized and resuspended in serum-free medium. Subsequently, 1 × 10^4^ cells were added to the upper chamber, while 500 μL of complete medium containing chemerin with or without PD98059 was placed in the lower chamber. After 12 h, the cells that failed to migrate from the upper chamber were removed with cotton swabs. Then, the migrated cells on the lower side of the membrane were photographed after being stained with 1% crystal violet (Mengbio, Chongqing, China).

### Statistical Analysis

The results are presented as the mean ± standard error of the mean (SEM), median or percentages. Unpaired t-tests were used for comparisons between two groups. One-way ANOVA followed by the LSD test was used to assess statistical significance among groups. The diagnostic value of plasma chemerin for PAH was evaluated by receiver operating characteristic (ROC) curve analysis. A value of *p* < 0.05 was considered statistically significant.

## Results

### Chemerin and CMKLR1 Were Upregulated in the Lung Tissues of PH Rats

**FIGURE 1:**
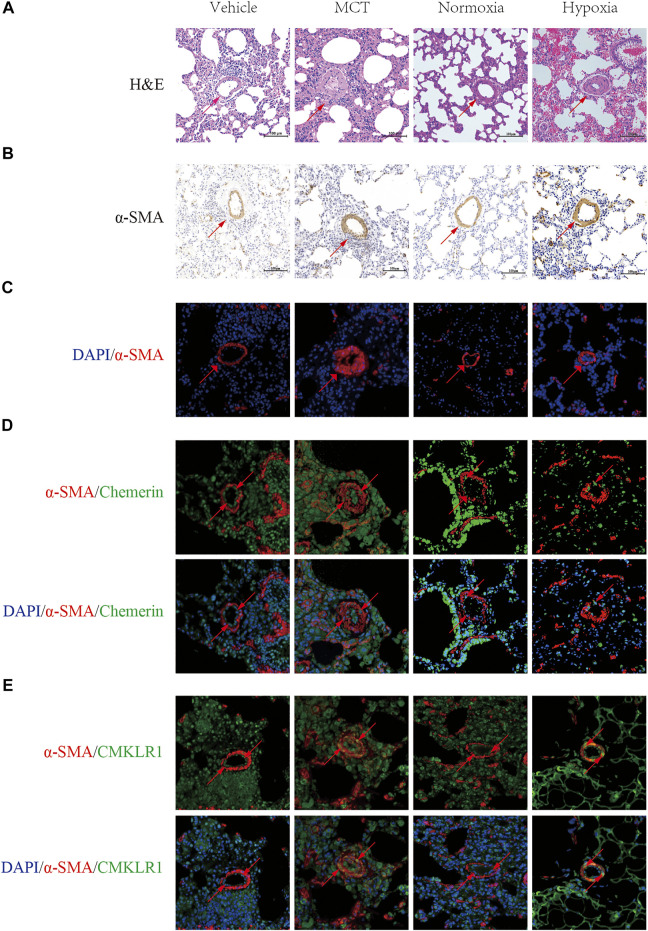
Localization and expression of Chemerin and its receptor CMKLR1 in rat lungs. Characterizing the localization of chemerin and its receptor CMKLR1 expression in rat lung tissues. **(A)** Representative images of H&E-stained lung tissues (original magnification, × 200, scale bars, 100 um). **(B)** Immunohistochemistry staining of pulmonary arteries stained with α-SMA (original magnification, × 200, scale bars, 100 um). **(C)** Immunofluoresce staining of pulmonary arteries stained with α-SMA (red) and DAPI (blue) (original magnification, × 400, scale bars, 50 um). **(D)** Representative immunofluorescence images of chemerin (green), α-SMA (red) and DAPI (blue) in normal and PH rat lung tissues (original magnification, × 400, Scale bars, 50 um). **(E)** Representative immunofluorescence images of CMKLR1(green), α-SMA (red) and DAPI (blue) in normal and PH rat lung tissues (original magnification, × 400, Scale bars, 50 um).

To evaluate the PH rat model, HE staining showed that the walls of pulmonary arterioles were significantly thickened and that the lumens were obviously narrowed in PH rats (Figure 1A). Moreover, Immunohistochemistry and Immunofluorescent staining of pulmonary arteries stained with α-SMA were used to further evaluate the PH model, and indicated that the walls of PH groups’ pulmonary arterioles were significantly thickened (Figures 1B,C). These data suggested that the PH rat models were successfully established by MCT administration and hypoxia.

Next, to localize and identify chemerin and its receptor CMKLR1, we co-immunostained lung tissues, with either antibody against chemerin and its receptor CMKLR1 together with a smooth muscle-specific marker, the α-smooth muscle actin. In control rat lungs, microscopic analysis revealed a strong staining of chemerin and its receptor CMKLR1 within the intimal layer (Figures 1D,E). However, as illustrated in Figures 1D,E, chemerin/CMKLR1 was upregulated within the media of PH rat lungs compared to the controls.

**FIGURE 2:**
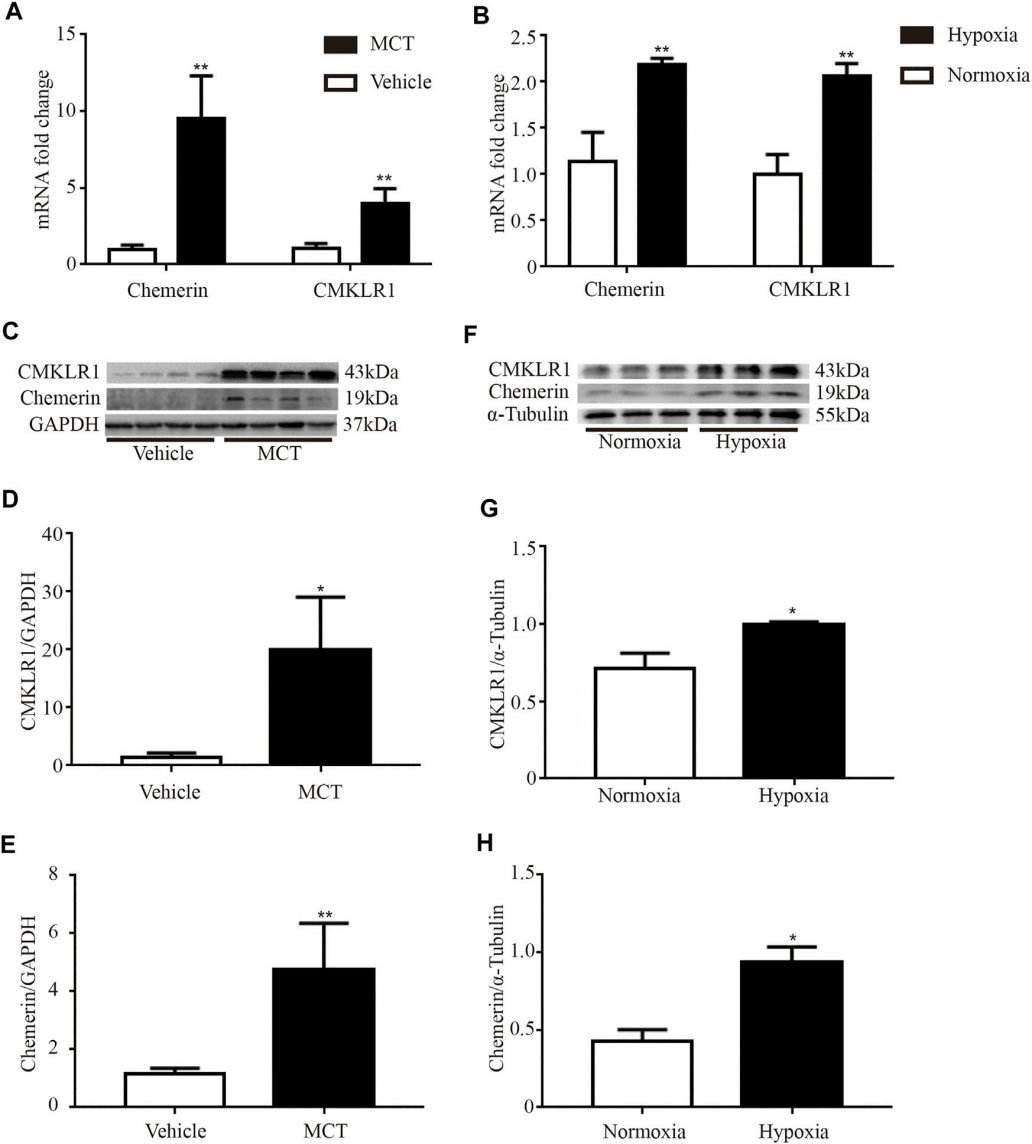
The mRNA and protein expression levels of chemerin and CMKLR1 are upregulated in MCT/hypoxia induced PH rat lungs. The mRNA and protein expression levels of chemerin and CMKLR1 in rat lungs. **(A)** The mRNA expression levels of chemerin and CMKLR1 in MCT group (*n* = 4 per group, **p < 0.01 *vs.* control group). **(B)** The mRNA expression levels of chemerin and CMKLR1 in hypoxia group (*n* = 3 in each group, **p < 0.01 *vs*. control group). **(C–E)** Western blot analysis of chemerin **(C and E)** and CMKLR1**(C and D)** expressions in the lungs of MCT group (*n* = 4 per group, *p < 0.05 *vs*. control group; **p < 0.01 *vs*. control group). **(F–H)** Western blot analysis of chemerin **(F and H)** and CMKLR1**(F and G)** expressions in the lungs of hypoxia group (*n* = 3 per group, *p < 0.05 *vs*. control group).

Then, the mRNA and protein expression levels of chemerin and CMKLR1 were measured in the rat lungs. As shown in Figure 2A, the mRNA levels of chemerin and CMKLR1 in the MCT group were significantly higher than those in the control group (both *p* < 0.01, Figure 2A), and these elevations were also verified by Western blot analysis (chemerin, *p* < 0.01, Figures 2C,E; CMKLR1, *p* < 0.05, Figures 2C,D). We also observed this phenomenon in the hypoxia group (both *p* < 0.01, Figure 2B; chemerin, *p* < 0.05, Figures 2F,H; CMKLR1, *p* < 0.05, Figures 2F,G).

### Hypoxia Upregulated CMKLR1 Expression in PASMCs

**FIGURE 3:**
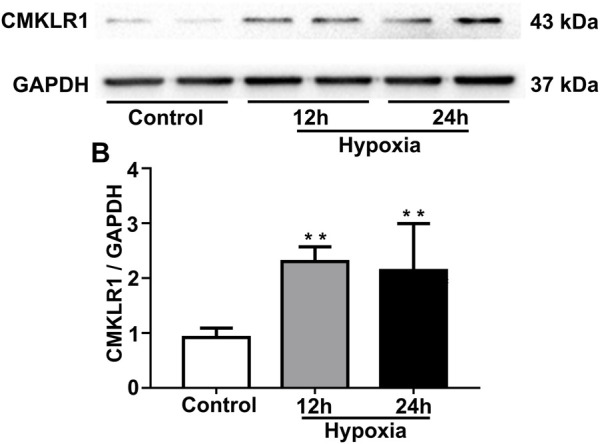
CMKLR1 expression is upregulated in PASMCs under hypoxic conditions. To mimic PH *in vitro*, PASMCs were exposed to hypoxia (2%) for two different durations (12 and 24 h). **(A,B)** Western blot analysis of CMKLR1 expression in the groups. **p < 0.01 vs. the control group.

To mimic PH conditions *in vitro*, PASMCs were exposed to hypoxia (2%) for 12 and 24 h, respectively. As shown in Figure 3, the protein level of CMKLR1 was markedly increased in the hypoxia groups compared to the control group (both *p*<0.01).

### Chemerin/CMKLR1 Promoted the Proliferation and Migration of PASMCs

**FIGURE 4:**
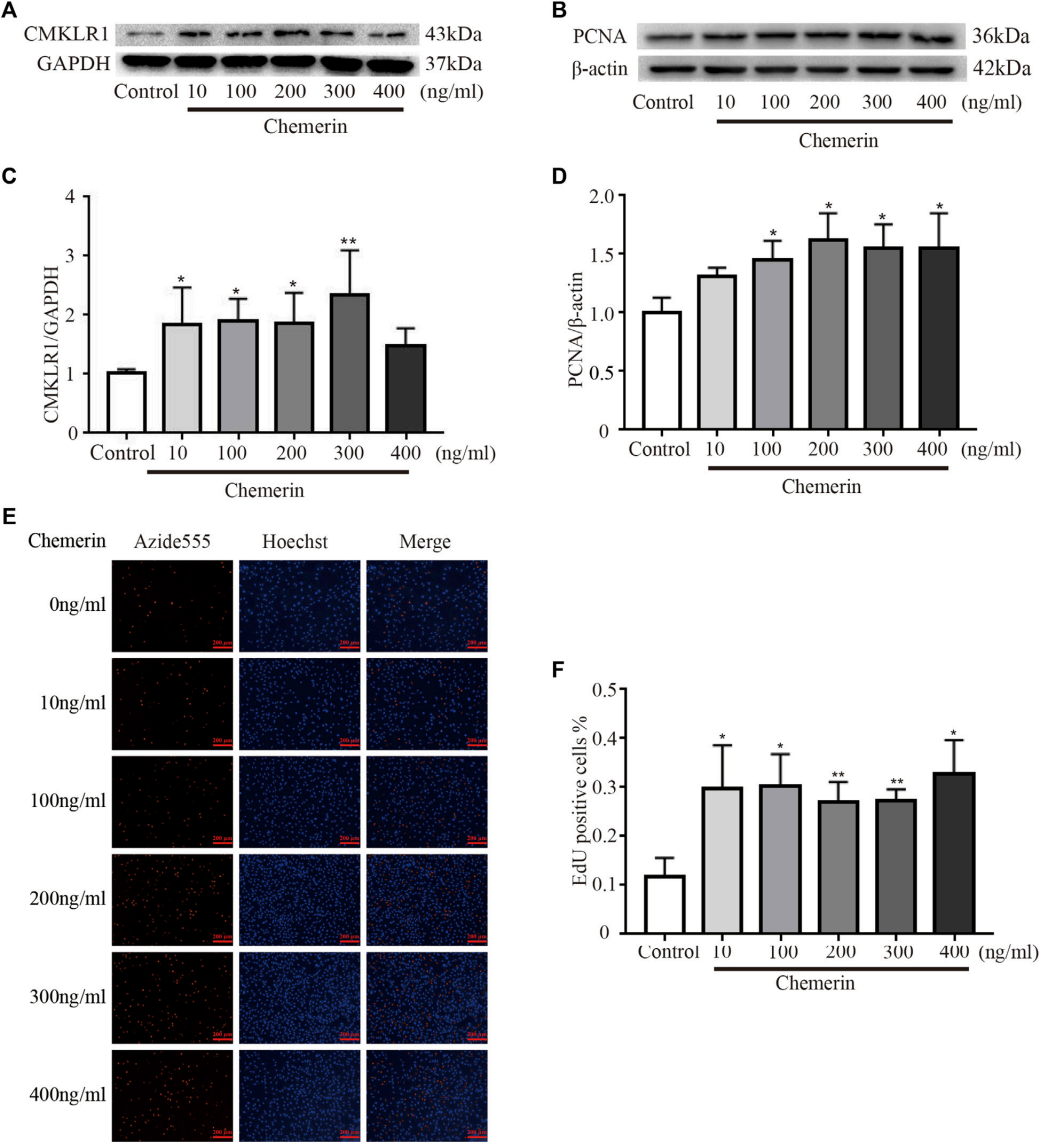
Chemerin/CMKLR1 promotes the proliferation of PASMCs. PASMCs were stimulated with various concentrations of chemerin. **(A–D)** Western blot analysis of the expression of CMKLR1 **(A and C)** and PCNA **(B and D)** in PASMCs after 24 h of treatment. **(E and F)** PASMCs proliferation was measured by an EdU staining assay after 24 h of treatment. (original magnification, ×100, scale bars, 200 μm). *p<0.05 *vs.* the control group; **p<0.01 *vs.* the control group.

PASMCs were treated with various concentrations of exogenous recombinant chemerin protein. As shown in Figures 4A,C, after stimulation for 24 h, the expression of CMKLR1 was significantly increased in the chemerin groups (10–300 ng/ml) compared to the control group (10 ng/ml, 100 ng/ml, and 200 ng/ml, all *p*<0.05; 300 ng/ml, *p* < 0.01). Furthermore, as the concentration of chemerin increased, the protein expression of PCNA was gradually upregulated compared to that in the control group (100 ng/ml, 200 ng/ml, 300 ng/ml, and 400 ng/ml, all *p* < 0.05, Figures 4B,D). To further elucidate whether the chemerin promotes the proliferation of PASMCs, we conducted the EdU assay. As shown in Figures 4E,F, chemerin can significantly promote the proliferation of PASMCs measured by EdU compared to control group (10 ng/ml, 100 ng/ml, 400 ng/ml, all *p* < 0.05, 200 ng/ml, 300 ng/ml, *p* < 0.01).

**FIGURE 5:**
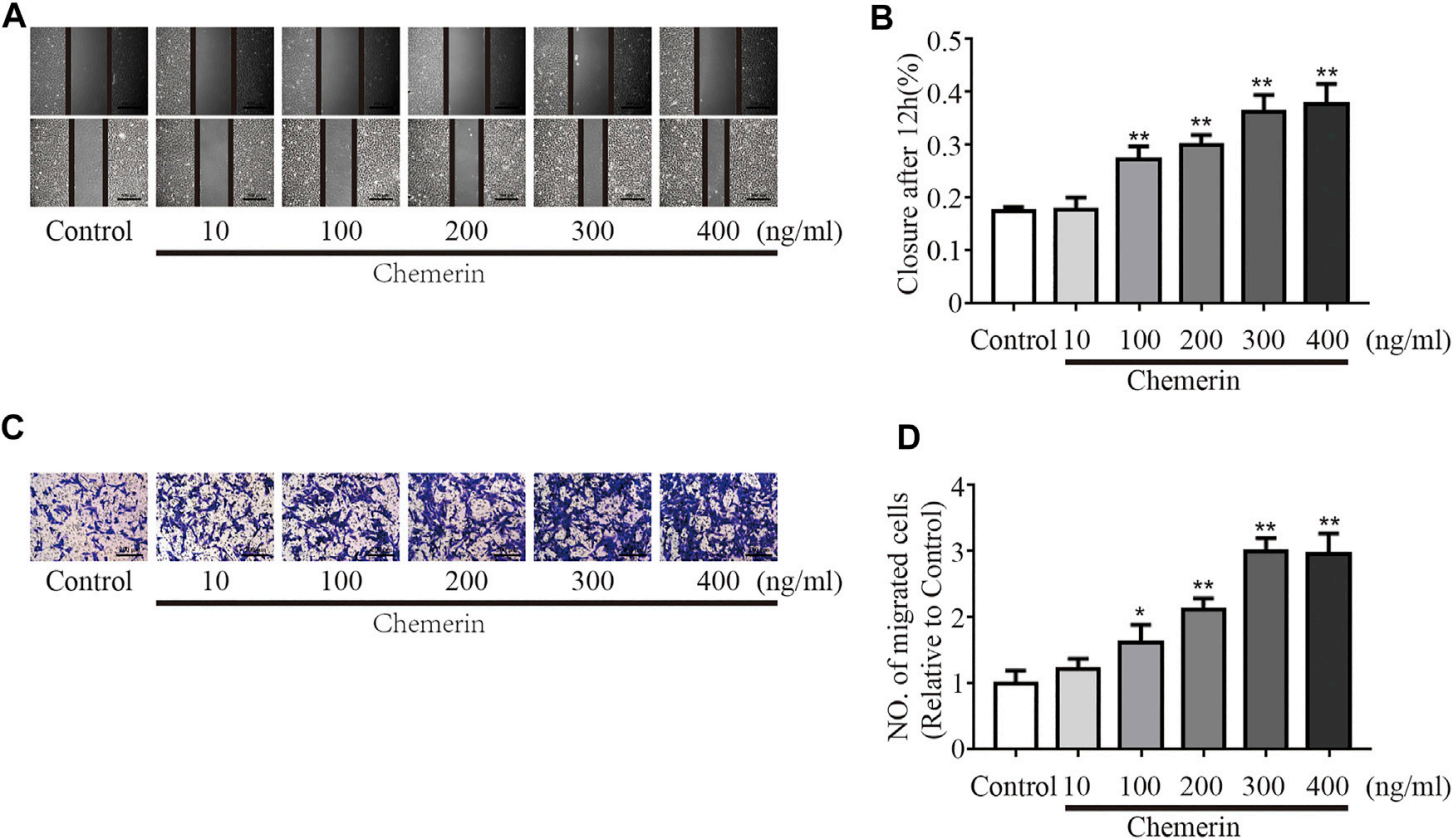
Chemerin/CMKLR1 promotes the migration of PASMCs. PASMCs were stimulated with various concentrations of chemerin. **(A–B)** Images of scratches in PASMCs photographed at 0 and 12 h under different treatment conditions (original magnification, × 50, scale bars, 500 μm). **(C–D)**: Transwell migration assays of PASMCs after different treatments for 12 h (original magnification, × 100, scale bars, 200 μm). *p < 0.05 vs. the control group; **p < 0.01 vs. the control group.

As shown in Figures 5A,B, as the concentration of chemerin increased, the migration distance of PASMCs gradually increased (100 ng/ml, 200 ng/ml, 300 ng/ml, and 400 ng/ml, all *p* < 0.01, Figures 5A,B). Consistent with the scratch assay results, the number of migrated cells was similarly increased by chemerin stimulation, as shown in the Transwell migration assay (100 ng/ml, *p* < 0.05; 200 ng/ml, 300 ng/ml, and 400 ng/ml, all *p* < 0.01, Figures 5C,D).

### Chemerin Mediated PASMCs Proliferation and Migration by Activating the ERK1/2 Signaling Pathway

**FIGURE 6:**
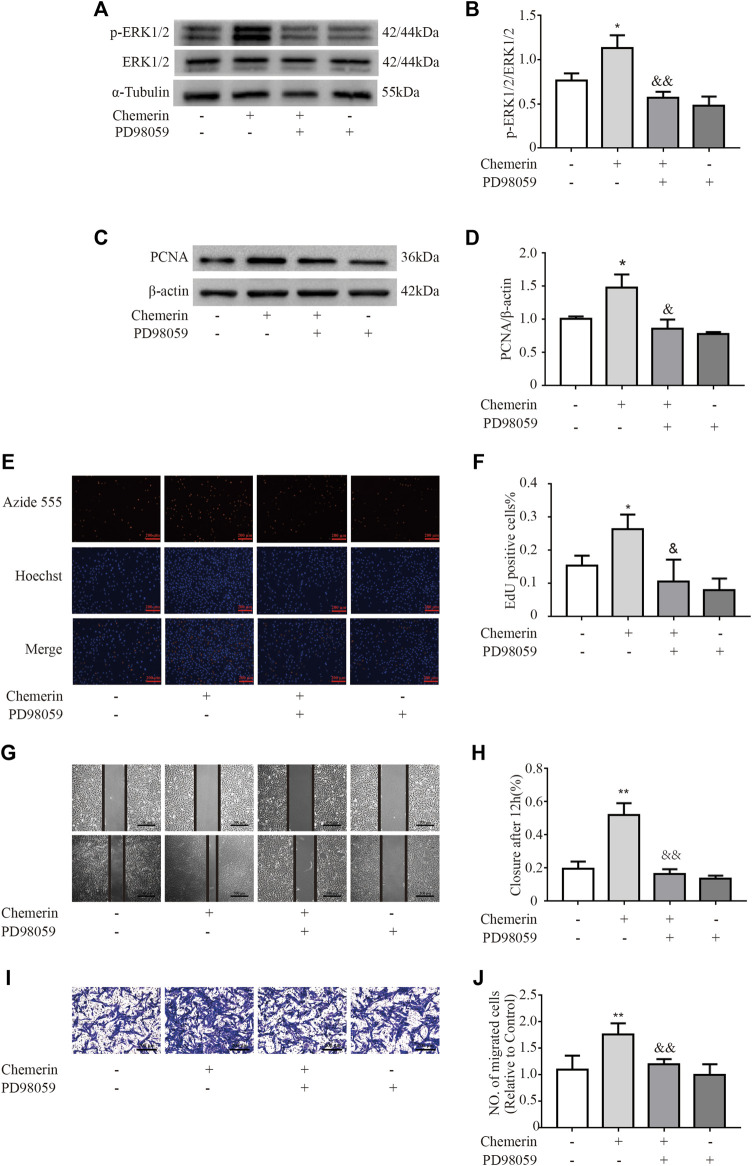
Chemerin induces phenotypic changes in PASMCs *via* the ERK1/2 signaling pathway. PASMCs were pretreated with PD98059 (50 µM) 30 min prior to chemerin treatment (300 ng/ml). **(A and B)** Western blot analysis of p-ERK1/2 and ERK1/2 expression in PASMCs after stimulation by chemerin with or without PD98059 pretreatment for 12 h. **(C and D)** Western blot analysis of PCNA expression in chemerin-treated cells with or without PD98059 pretreatment for 24 h. **(E and F)** PASMC cell proliferation was measured by an EdU staining assay after 24 h of treatment (original magnification, × 100, scale bars, 200 μm). **(G and H)** Images of scratches in PASMCs photographed at 0 and 12 h under different treatment conditions (original magnification, × 50, scale bars, 500 μm). **(I and J)** Transwell migration assays of PASMCs after different treatments for 12 h (original magnification, ×100, scale bars, 200 μm). *p < 0.05 vs. the control group; **p < 0.01 *vs.* the control group; & *p* < 0.05 *vs*. the chemerin group. && *p* < 0.01 *vs.* the chemerin group.

To investigate the potential mechanism of chemerin-induced phenotypic changes in PASMCs, the key regulatory proteins in the ERK1/2 signaling pathway were analyzed by Western blotting. As shown in Figures 6A,B, under exogenous chemerin stimulation, the ERK1/2 signaling pathway was significantly activated, as indicated by the increased p-ERK/ERK ratios in the chemerin group compared with the control group, and the increase was reduced by PD98059 (an inhibitor of ERK1/2 signaling) (*p < 0.05 *vs*. control group, &&*p* < 0.01 *vs*. chemerin group). Furthermore, PD98059 pretreatment eliminated the increase in PCNA expression in chemerin-treated PASMCs (*p < 0.05 *vs*. control group, &*p* < 0.05 *vs*. chemerin group, Figures 6C,D). Similarly, The proliferation promoting effect of chemerin on PASMCs can be decreased by PD98059 (*p < 0.05 *vs.* control group, &*p* < 0.05 *vs*. chemerin group, Figures 6E,F). In addition, the migration assays showed that chemerin induced sharp increases in the migration distance and number of migrated PASMCs, which were reversed by PD98059 (**p < 0.01 vs. control group, &&P < 0.01 *vs*. chemerin group, Figures 6G,H and Figures 6I,J).

### Plasma Chemerin Levels Were Significantly Upregulated in IPAH Patients

The clinical characteristics of all participants were summarized in Table 1. The median age of control individuals was 25 years and that of IPAH patients was 31.5 years. The proportion of females was 85.7% in the IPAH group, and 61.9% in the control group. The median mPAP was 53 mmHg, the median PAWP was 7.5 mmHg, the median PVR was 13.06 Wood units, and median cardiac output (CO) was 3.85 L/min in the IPAH group.

**TABLE 1:** Basic demographics of all participants in clinical trials. The mPAP (mean pulmonary arterial pressure), PAWP (pulmonary artery wedge pressure) and PVR (pulmonary vascular resistance) and CO (cardiac output) were measured by the right cardiac catheterization (RHC). The Treatment means that the patient has been treated with targeted drugs for IPAH (idiopathic pulmonary arterial hypertension), before the RHC for the first time at the First Affiliated Hospital of Chongqing Medical University.

**Baseline demographics**	**Participants**	
	**Control (*n* = 21)**	**IPAH (*n* = 14)**
Age (year)		
Mean (SEM)	25.67 (0.78)	29.5 (2.72)
Median (range)	25 (23–36)	31.5 (10–52)
Sex		
Female, % (n)	61.9% (13)	85.7% (12)
Male, % (n)	38.1% (8)	14.3% (2)
mPAP (mmHg)		
Mean (SEM)	_	55.57 (4.20)
Median (range)	_	53 (27–90)
PAWP (mmHg)		
Mean (SEM)	_	7.71 (1.11)
Median (range)	_	7.5 (0–14)
PVR (Woods units)		
Mean (SEM)	_	14.44 (2.77)
Median (range)	_	13.06 (3.62–42)
CO (L/min)		
Mean (SEM)	_	4.26 (0.45)
Median (range)	_	3.85 (1.5–7.2)

https://www.jianguoyun.com/p/DQIvgQQQlJrqCRi2iqYE

**FIGURE 7:**
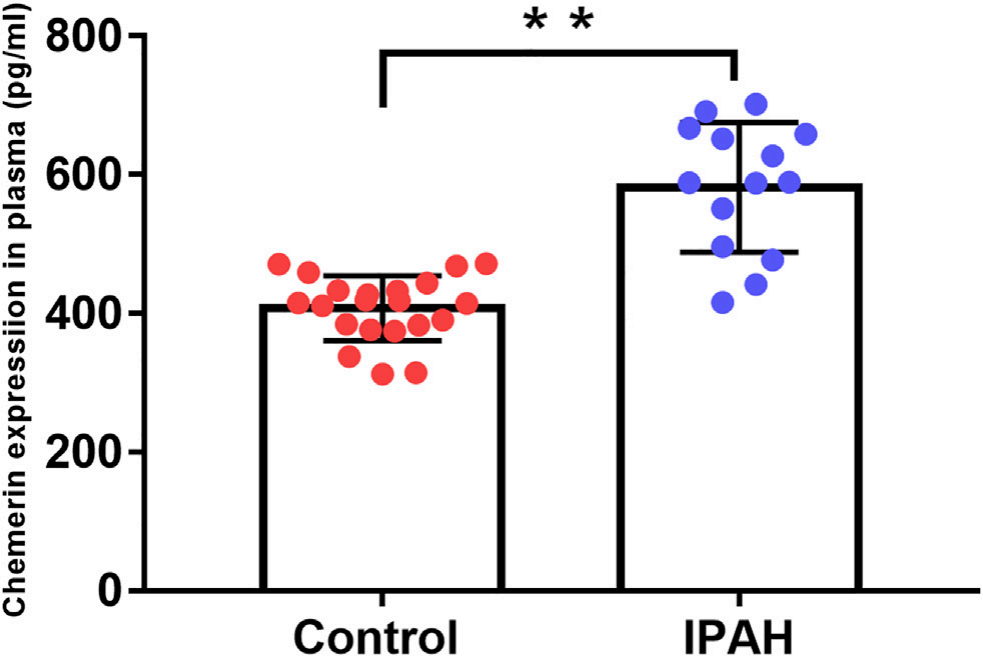
Plasma chemerin levels are increased in IPAH patients. Human plasma chemerin protein levels were measured by ELISA in the control and IPAH groups. Quantitative analysis of chemerin protein expression in the plasma of each group was also performed (control, *n* = 21; IPAH, *n* = 14). **p < 0.01 *vs.* the control group.

ELISA assay result showed that compared to the control group, the plasma level of chemerin was markedly increased in the IPAH group (581.7 ± 24.9 pg/ml *vs*. 407.5 ± 10.23 pg/ml, *p* < 0.01, Figure 7).

### Diagnostic Value of Chemerin Expression in IPAH

**FIGURE 8:**
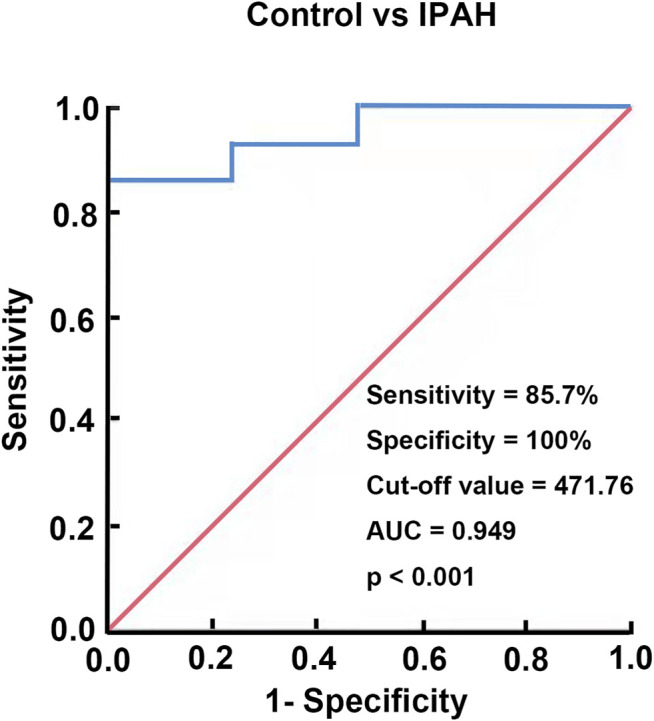
Diagnostic value of chemerin expression in IPAH. A ROC curve of plasma chemerin for discriminating IPAH patients from healthy controls.

To assess the diagnostic value of plasma chemerin, we generated a ROC curve using the expression data measured by ELISA. As shown in Figure 8, the area under the ROC curve (AUC) reached 0.949 [95% confidence interval (CI): 87.3–100%], and plasma chemerin had a satisfactory sensitivity (85.7%) and specificity (100%) at a concentration of 471.76 pg/ml (*p*<0.001), which indicated considerable diagnostic value for IPAH.

## Discussion

The present study revealed that chemerin/CMKLR1 promoted the proliferation and migration of PASMCs via activating the ERK1/2 signaling pathway in PH rats and the plasma level of chemerin was markedly increased in iPAH patients. The main findings are as follows: 1) the expression of chemerin and CMKLR1 was upregulated in the lungs of MCT/hypoxia induced PH rats; 2) CMKLR1 expression was significantly increased in PASMCs under hypoxic conditions; 3) PASMC stimulation with exogenous chemerin promoted cell proliferation and migration via the ERK1/2 signaling pathway; and 4) the expression of chemerin protein was significantly increased in plasma of IPAH patients. Increasing evidence has shown that chemerin is closely associated with cardiovascular diseases; in addition to regulating systemic blood pressure and promoting intimal hyperplasia, chemerin contributes to angiogenesis and vascular remodeling in hypertension (Kunimoto et al., 2015; Xiong et al., 2016; Liu et al., 2019; Wen et al., 2019). Besides, chemerin can enhance vascular responses to vasoconstrictors in the pulmonary artery and reduce acetylcholine-induced pulmonary artery vasodilation partly by nitric oxide (NO) signaling and oxidative stress (Hanthazi et al., 2019). Furthermore, as an active fragment of chemerin, chemerin-9 augmented the contraction of isolated pulmonary arteries from MCT rats at least partially through enhancing CMKLR1 activity in smooth muscle (Omori et al., 2020). In this study, for the first time, we revealed that chemerin was significantly upregulated in the lung tissues of MCT/hypoxia induced PH rats, indicating that chemerin associated with PH.

CMKLR1, as a functional receptor, directly interacts with chemerin, contributing to various biological processes, such as inflammation, immune responses, adipogenesis and angiogenesis (Goralski et al., 2007; Nakamura et al., 2018; Pachynski et al., 2019; Xie et al., 2020). Omori A et al. reported that the protein levels of CMKLR1 were markedly increased in the lungs and PASMCs of MCT-induced PH rats (Omori et al., 2020). Similarly, in the present study, we showed that CMKLR1 was sharply upregulated in the lungs of MCT and hypoxia induced-PH rats and further demonstrated that the expression of CMKLR1 was significantly elevated in isolated PASMCs under hypoxic conditions.

Excessive PASMC proliferation and migration play crucial roles in pulmonary vascular remodeling, thus facilitating PH development (Tuder 2017). A previous study demonstrated that silencing CMKLR1 could alleviate the proliferation of thoracic aortic smooth muscle cells (SMCs) in mice, which was associated with a decrease in p-JNK expression (Liu et al., 2016). Here, we showed that chemerin significantly upregulated CMKLR1 expression in PASMCs even at very low concentrations, indicating that the regulatory effects of chemerin on PASMCs may ultimately be mediated by CMKLR1. However, this finding was different from a recent study, which indicated that chemerin alone did not exert a positive effect on PASMCs proliferation (Hanthazi et al., 2020). These discrepant results may result from different cell treatment conditions and variable cell proliferation protocol. Nevertheless, other than CMKLR1, whether the other receptors of chemerin participate in theses signal transduction needs to be further investigated.

ERK1/2 signaling is a major member of the MAPK family, which is widely involved in many physiological and pathological processes (Tian et al., 2018; Bao et al., 2019; Hu et al., 2020; Zou et al., 2020). It has been demonstrated that the ERK1/2 signaling pathway is significantly activated in a variety of PH animal models, while knockdown of the ERK1/2 gene effectively suppresses pulmonary vascular remodeling, ultimately preventing PH development (Yu et al., 2017). The intrinsic links between chemerin and the ERK1/2 signaling pathway have been verified in a few pathological processes, including systemic hypertension, lipolytic metabolism and insulin resistance (Zhang et al., 2014; Fu et al., 2018; Jiang et al., 2018). Lobato et al. found that chemerin increased the contractile response of the aorta to phenylephrine (PE) and ET-1 by activating ERK1/2 (Lobato et al., 2012). Kunimoto H et al. reported that chemerin stimulated the proliferation and migration of SMCs via oxidative-dependent phosphorylation of ERK1/2 in hypertension (Kunimoto et al., 2015). Similarly, we found that chemerin could activate the ERK1/2 signaling pathway in PASMCs and PD98059 reversed the regulatory effects of chemerin on PASMCs. In addition, a previous study indicated that chemerin expression was elevated through ERK1/2 signaling in human coronary artery endothelial cells in response to hypoxia (Chua et al., 2016). These data suggest that there may be crosstalk between chemerin and ERK1/2 signaling under different pathological conditions.

Evidence from multiple clinical studies suggests that circulating chemerin levels are associated with cardiovascular diseases (Ji et al., 2014; Zhang et al., 2017; Wójcik et al., 2020)**.** On that basis, Zhou X et al. reported that serum chemerin levels could be used to predict the presence of adverse cardiovascular events in patients with chronic heart failure (Zhou et al., 2019). Interestingly, in our clinical study, we also found that chemerin expression was significantly upregulated in the plasma of IPAH patients, which was consistent with the results of the rat experiment mentioned previously. Moreover, ROC curve analysis indicated that plasma chemerin may have a considerable diagnostic value for IPAH as its high sensitivity and specificity with an AUC of 0.949. These data demonstrated that plasma chemerin level is closely related to PAH. However, a study with larger sample size of IPAH patients and long-term follow-up is needed to further confirm the potential association between chemerin plasma levels and PAH outcomes.

In conclusion, our study reveals that the chemerin/CMKLR1 axis promotes PASMC proliferation and migration by activating the ERK1/2 signaling pathway, and chemerin protein levels are increased in the plasma of IPAH patients. The present study provides significant insights intothe development of potential therapies for PH.

## Data Availability

The original contributions presented in the study are included in the article/Supplementary Material, further inquiries can be directed to the corresponding author.
